# Metabolite profiling with HPLC-ICP-MS as a tool for in vivo characterization of imaging probes

**DOI:** 10.1186/s41181-017-0037-5

**Published:** 2018-01-22

**Authors:** Eszter Boros, Omar R. Pinkhasov, Peter Caravan

**Affiliations:** 1 A. A. Martinos Center for Biomedical Imaging, Massachusetts General Hospital, Harvard Medical School, 149 13th Street, Suite 2301, Charlestown, MA 02129 USA; 2 Institute for Innovation in Imaging, Department of Radiology, Massachusetts General Hospital, Building 149, Room 2301, 13th Street, Charlestown, Boston, MA 02129 USA; 3 Present address: Department of Chemistry, Stony Brook University, 100 Nicolls road, Stony Brook, New York, NY 11790 USA

**Keywords:** Metabolite analysis, Preclinical studies, Radiometals, Hplc-Icp-ms

## Abstract

**Background:**

Current analytical methods for characterizing pharmacokinetic and metabolic properties of positron emission tomography (PET) and single photon emission computed tomography (SPECT) probes are limited. Alternative methods to study tracer metabolism are needed. The study objective was to assess the potential of high performance liquid chromatography - inductively coupled plasma - mass spectrometry (HPLC-ICP-MS) for quantification of molecular probe metabolism and pharmacokinetics using stable isotopes.

**Methods:**

Two known peptide-DOTA conjugates were chelated with ^nat^Ga and ^nat^In. Limit of detection of HPLC-ICP-MS for ^69^Ga and ^115^In was determined. Rats were administered 50–150 nmol of Ga- and/or In-labeled probes, blood was serially sampled, and plasma analyzed by HPLC-ICP-MS using both reverse phase and size exclusion chromatography.

**Results:**

The limits of detection were 0.16 pmol for ^115^In and 0.53 pmol for ^69^Ga. Metabolites as low as 0.001 %ID/g could be detected and transchelation products identified. Simultaneous administration of Ga- and In-labeled probes allowed the determination of pharmacokinetics and metabolism of both probes in a single animal.

**Conclusions:**

HPLC-ICP-MS is a robust, sensitive and radiation-free technique to characterize the pharmacokinetics and metabolism of imaging probes.

**Electronic supplementary material:**

The online version of this article (10.1186/s41181-017-0037-5) contains supplementary material, which is available to authorized users.

## Background

Biomedical imaging uses exogenous probe molecules that often contain metal ions, metalloids or halogens (Zeglis & Lewis 2011; Blower 2015). Characterization of probe pharmacokinetics and metabolic stability is critical for understanding in vivo performance and to develop improved probes. Current analytical methods for characterizing pharmacokinetic and metabolic properties of positron emission tomography (PET) and single photon emission computed tomography (SPECT) probes are limited. High-performance liquid chromatography (HPLC) with a radioactivity detector is plagued by poor sensitivity; relatively high amounts of radioactivity are required resulting in increased radiation exposure for operators and/or poor peak resolution due to large detector volumes (Roivainen et al. 2013; Beykan et al. 2016). Alternately, HPLC fractions can be collected and analyzed with a well counter for greater sensitivity, but this is time consuming and sacrifices resolution provided by continuous radio-detection. HPLC-mass spectrometry methods can be used with non-radioactive probes, but these involve considerable method development for each probe while the high background of other ionizable components in the biological matrix impedes sensitivity (Simon-Manso et al. 2013; Imbert et al. 2014).

HPLC – inductively coupled plasma mass spectrometry (HPLC-ICP-MS) offers a potential solution to these problems. The extremely high sensitivity of ICP-MS allows for the detection of low quantities of probe and probe metabolites similar to those employed in nuclear medicine (Kotrebai et al. 2000; Jabłońska-Czapla et al. 2014). The high sensitivity of HPLC-ICP-MS allows evaluation of PET and SPECT probes by using their corresponding stable isotopes. Different methods of chromatographic separation (e.g. reverse phase, size exclusion) provide metabolite and pharmacokinetic analysis of both low molecular weight and protein-associated metabolites, while completely eliminating radiation exposure. Furthermore, the mass analyzer scan rate of ICP-MS is fast enough such that multiple elements can be analyzed offering the potential of assessing multiple probes with different elemental labels. Therefore this method can serve as a simple way to simultaneously screen promising lead compounds without the use of radioactive isotope or a radiochemistry laboratory.

^64^Cu-FBP2 and ^64^Cu-FBP3 are peptide-DOTA conjugates that differ in peptide structure and were previously evaluated as PET imaging probes for arterial thrombosis (Ciesienski et al. 2013). ^64^Cu-FBP3 was rapidly metabolized with peptide degradation in vivo, while ^64^Cu-FBP2 was relatively stable. Here we complexed each of these peptide-DOTA conjugates with Ga and In to give 4 compounds. We expected to observe differences in peptide metabolism and also anticipated that the Ga derivatives would show some transchelation to transferrin or other blood proteins (Dumont et al. 2011; Blasi et al. 2014). With these four compounds we asked: Could HPLC-ICP-MS detect probe concentrations in the range observed in nuclear medicine? Could we distinguish and quantify intact probe, metabolites, and transchelation products in ex vivo blood samples? Could we assess the pharmacokinetic and metabolic behavior of Ga- and In-labeled compounds administered simultaneously?

## Methods

### Materials

**Fig. 1:**
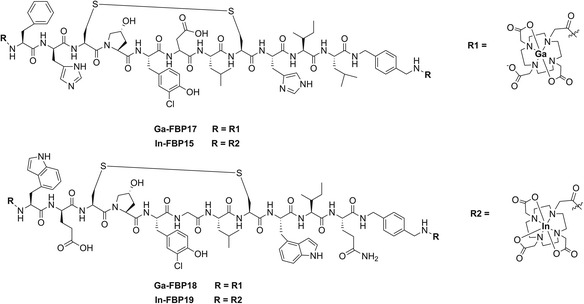
Chemical structures of In-FBP15, Ga-FBP17, Ga-FBP18 and In-FBP19

^*nat*^*Ga*_*2*_*FBP17.* fbp2 ligand (20 mg, 8.7 μmol) was mixed with a solution of Ga(NO_3_)_3_ • 6H_2_O (3 eq.) and the pH of the solution was adjusted slowly to 7.4 and stirred for 18 h. The resulting turbid solution was filtered and purified using preparative HPLC to isolate the bis-gallium complex (8 mg, 3.3 μmol, 37% yield) eluting at 15.3 min. Fractions containing pure complex were pooled, neutralized and lyophilized immediately to prevent complex dissociation. Absence of non-chelated Ga species was assessed using HPLC-ICP-MS. Theoretical M_W_ for C_103_H_142_ClGa_2_N_25_O_29_S_2_: 2432.81. Observed: 1217.9 [M + 2H^+^]^2+^.

^*nat*^*In*_*2*_*FBP15.* fbp2 ligand (20 mg, 8.7 μmol) was mixed with a solution of InCl_3_ (3 eq.) and the pH of the solution was adjusted slowly to 7.4 and stirred for 18 h. The resulting turbid solution was filtered and purified using preparative HPLC to isolate the bis-indium complex (6 mg, 2.4 μmol, 28% yield) eluting at 16.1 min. Fractions containing pure complex were pooled, neutralized and lyophilized immediately to prevent complex dissociation. Absence of non-chelated In species was assessed using HPLC-ICP-MS. Theoretical M_W_ for C_103_H_142_ClIn_2_N_25_O_29_S_2_: 2522.76. Observed 1262.9 [M + 2H^+^]^2+^.

^*nat*^*Ga*_*2*_*FBP18.* fbp3 ligand (20 mg, 8.5 μmol) was mixed with a solution of Ga(NO_3_)_3_ • 6H_2_O (3 eq.) and the pH of the solution was adjusted slowly to 7.4 and stirred for 18 h. The resulting turbid solution was filtered and purified using preparative HPLC to isolate the bis-gallium complex (4 mg, 1.6 μmol, 18% yield) eluting at 15.7 min. Fractions containing pure complex were pooled, neutralized and lyophilized immediately to prevent complex dissociation. Absence of non-chelated Ga species was assessed using HPLC-ICP-MS. Theoretical M_W_ for C_106_H_141_ClGa_2_N_24_O_30_S_2_: 2513.77. observed 1257.3 [M + 2H^+^] ^2+^.

^*nat*^*In*_*2*_*FBP19.* fbp3 ligand (20 mg, 8.5 μmol) was mixed with a solution of InCl_3_ (3 eq.) and the pH of the solution was adjusted slowly to 7.4 and stirred for 18 h. The resulting turbid solution was filtered and purified using preparative HPLC to isolate the bis-indium complex (6 mg, 2.3 μmol, 27% yield) eluting at 15.2 min. Fractions containing pure complex were pooled, neutralized and lyophilized immediately to prevent complex dissociation. Absence of non-chelated In species was assessed using HPLC-ICP-MS. Theoretical M_W_ for C_106_H_141_ClIn_2_N_24_O_30_S_2_: 2559.75. Observed 1280.8 [M + 2H^+^]^2+^.

Ga(DOTA), Tm(DOTP), Cu(cyclam), and Zr(DFO) were prepared using literature protocols. (Viola et al. 2006; Deri et al. 2014; Tasker & Sklar 1975) In brief, 1.5 equivalents of ligand were treated with 1 equivalent of the corresponding metal chloride salt at pH 2.5. Subsequently the pH was increased slowly to 7.4 using 0.1 M NaOH. Iohexol was obtained from Bracco S.p.A. The peptide-DOTA conjugate precursors (Fig. 1) fbp2 and fbp3 were synthesized as previously described (Ciesienski et al. 2013). Purification methods encompassed preparative HPLC purification using a Varian Prostar system with two Prostar 210 pumps and a Prostar 325 UV/Vis detector, using a Phenomenex Luna C18 column (250 × 21.2 mm, 10 μm) using method 1: A-H_2_O with 0.1% trifluoroacetic acid (TFA) and as mobile phase B-CH_3_CN with 0.1% TFA. Flow rate of 15 mL/min, 5%B isocratic 0–5 min; 5 to 30% B, 5–11 min; 30 to 75% B, 11–20 min; 75 to 95% B, 20–23 min; 95% B isocratic, 23–27 min. Liquid chromatography-electrospray mass spectrometry (LC-MS) HPLC purity analyses (both UV and MS detection) were carried out on an Agilent 1260 system, using a Phenomenex Luna C18(2) column: 100 mm × 2 mm, 0.8 mL/min flow rate) with UV detection at 220, 254, and 280 nm and +ESI using method 2: A-H_2_O with 0.1% trifluoroacetic acid (TFA) and as mobile phase B-CH_3_CN with 0.1% TFA. Flow rate 0.7 mL/min, 0–10 min, 5 to 95% B. fbp2 and fbp3 peptide-DOTA conjugates were synthesized as previously described (Ciesienski et al. 2013; Oliveira et al. 2015).

Ga and In transferrin complexes were synthesized using a 1.3 equivalent excess of protein and purification with Zeba™ Spin Desalting Columns (ThermoFisher Scientific), to remove unchelated Ga and In. TBS buffer was prepared by mixing 150 mM NaCl, 50 mM Tris and 5 mM NaHCO_3_ followed by stirring over chelex overnight and subsequent filtration.

### Analytical method development, detection limit and quantification

An Agilent 8800 ICP-MSMS interfaced to an Agilent 1260 HPLC was used. 20% option gas (20% O_2_, 80% Ar) was added to pyrolyze any carbon deposits on the cones from the HPLC mobile phase while 40% O_2_ was used as cell gas when conducting reverse phase analysis. HPLC method 3: C18 column (Kromasil 5 μm C18, 250 × 4.60 mm), 0.8 mL/min, mobile phase A (water, 10 mM NaOAc), mobile phase B (90% acetonitrile, 10% 10 mM NaOAc), gradient: min. 0–2: 5% B; min 12: 50% B; min 13: 95% B; min 15: 95% B; min 16: 5% B; min 18: 5% B. HPLC method 4: size exclusion chromatography (SEC) Column (Phenomenex BioSep-SEC-s2000, 300 × 7.80 mm), 1 mL/min, mobile phase TBS, isocratic, 18 min. The SEC column was standardized using a protein marker kit (Sigma-Aldrich, P/N: MWGF1000). Ga(DOTA) and In(DOTA) were used to determine the limit of detection and limit of quantification, and to generate a calibration curve for quantification using RP-separation. Calibration standards of Ga(DOTA) and In(DOTA) ranging from 10 nM to 1 μM were prepared by serial dilution from stocks. 100 μL of each standard was analyzed by direct injection onto the reverse phase HPLC-ICP-MS (HPLC method 3), and the integrals of each metal-FBP peak were recorded. An aliquot of each calibration standard was then digested 1:1 with nitric acid and incubated overnight at 37 °C before being analyzed for its metal concentration by ICP-MS. These metal concentrations were plotted against the previously recorded integrals to generate the metal-FBP calibration plots (Additional file 1: Figure S1). Limits of detection and quantification were calculated using an established method (MacDougall & Crummett 1980).

### In vivo experiment

Ga-FBP17, Ga-FBP18, In-FBP15, In-FBP19 (50–150 nmol, with concentrations determined by direct inject ICP-MS) in 0.6 mL sterile PBS were injected either individually or as a mixture of Ga-FBP17/In-FBP15 or Ga-FBP18/In-FBP19 into anesthetized rats via cannulated femoral vein, followed by saline flush. Blood (0.2 mL) was drawn at time points 5, 10, 15, 30 and 60 min post injection via cannulated femoral artery into heparin-containing tubes. Tubes were centrifuged at 2000 g for 10 min to separate the plasma. Plasma was diluted 1:1 with chelex-treated TBS and 100 μL of sample was injected onto the HPLC-ICP-MSMS, scanning for Ga and In at m/z = 69 and 115, respectively. Peaks were integrated and measured against a standard calibration curve.

## Results

### Multi-element detection capability

**Fig. 2:**
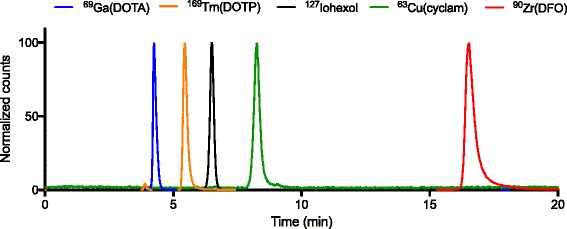
Example of simultaneous multi-element HPLC-ICP-MS analysis of a mixture of different compounds used in imaging applications at approximate compound concentrations of 1 μM. Data is normalized to the most dilute sample to be represented on the same scale

To test the capability of HPLC-ICP-MS for simultaneous detection of different elements from the same sample without significant matrix interference, we carried out simultaneous detection of Ga, Tm, I, Cu and Zr from a single injection mixture of Ga(DOTA), Tm(DOTP), Iohexol, Cu(cyclam), and Zr(DFO). Figure 2 exemplifies the simultaneous detection of different elements relevant for imaging applications from a single injection of a mixture.

### Limit of detection and quantification

Additional file 1: Figure S1 shows calibration curves for ^69^Ga and ^115^In obtained with Ga(DOTA) and In(DOTA) using reverse phase chromatography. The limit of detection was 0.16 pmol for ^115^In and 0.53 pmol for ^69^Ga, while the limit of quantification was 0.46 pmol for ^115^In and 0.89 pmol for ^69^Ga (Additional file 1: Table S1).

### Reverse phase chromatography analysis of plasma samples

**Fig. 3:**
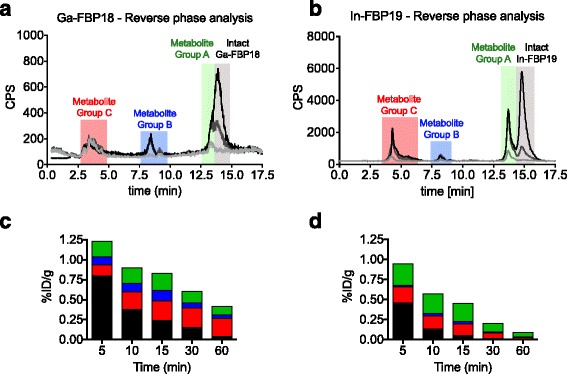
Overlaid HPLC-ICP-MS chromatograms of Ga-FBP18 (**a**) and In-FBP19 (**b**) for 5 min (black), 15 min (dark grey) and 60 min (light grey) post injection. Identified intact complex and metabolite groups are denoted in color. Panels C and D: Quantification of total probe and metabolite groups as a function of time post injection for Ga-FBP18 (**c**) and In-FBP19 (**d**)

Figure 3a, b shows example traces for blood samples drawn 5, 15 and 60 min post injection of either Ga-FBP18 (A) or In-FBP19 (B) obtained with a C18 column. Both compounds underwent rapid metabolism with at least 4 metabolites readily apparent on the HPLC-ICP-MS chromatograms. On the other hand, Ga-FBP17 and In-FBP15 showed only minimal degradation over time (Additional file 1: Figure S3). The observed metabolic behavior is comparable to what was observed previously for the ^64^Cu labeled probes (Ciesienski et al. 2013; Oliveira et al. 2015). For simplicity we assigned the metabolites to one of three groups based on their retention times. Figure 3c and d show stacked bar plots as a function of time for Ga-FBP18 and In-FBP19, respectively. The height of the bar gives the total metal concentration in the plasma expressed as percent of injected dose per gram of plasma (%ID/g), and the different colors represent the concentrations of the intact probe and the different metabolite groups. The amount of intact probe as a percentage of total metal ion concentration decreases with time post injection. For Ga-FBP18 the relative amount of metabolite group C (Fig. 3c, Red) increased with time post injection. We speculate that these metabolites, which elute with the shortest retention times are low molecular weight polar fragments. On the other hand the relative amounts of the In-FBP19 metabolites are roughly constant with time suggesting that the rate of metabolism is similar to the rate of clearance of the metabolites.

**Fig. 4:**
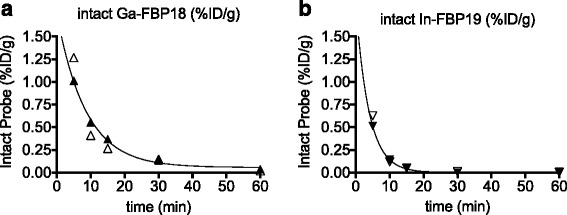
Quantification of intact Ga-FBP18 (**a**) and In-FBP19 (**b**) based on reverse phase HPLC-ICP-MS from single compound injection (filled triangles) and co-injection (open triangles). Solid line is monoexponential fit to the data

Figure 4 shows the concentrations of intact Ga-FBP18 (A) and In-FBP19 (B) as a function of time (see Additional file 1: Figure S4 for Ga-FBP17 and In-FBP15). Assuming a monoexponential clearance, the calculated half-lives of the intact complexes were 9.0 min (Ga-FBP17), 4.9 min (In-FBP15), 5.9 min (Ga-FBP18) and 2.7 min (In-FBP19).

### Analysis of co-injected compounds

We co-administered Ga-FBP17 with In-FBP15 and Ga-FBP18 with In-FBP19. In both cases, the pharmacokinetics and metabolite profile for the Ga- and In-probes administered to mice as a mixture were similar to that measured for the compounds administered individually. This is demonstrated in Fig. 4 and Additional file 1: Figure S7, where the concentrations represented by the filled symbols indicate data from a single injection and open symbols data from a co-injection.

### Size exclusion chromatography analysis of plasma samples

**Fig. 5:**
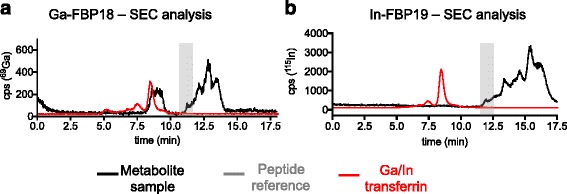
SEC HPLC-ICP-MS chromatograms of Ga-FBP18 plasma sample 15 min post injection (black) and pure Ga-transferrin (red) (**a**) or In-FBP19 plasma sample 15 min post injection (black) and In-transferrin (red) (**b**). Elution time of intact complex is indicated by grey box

In addition to reverse phase separation, samples were analyzed using SEC to detect transchelation events to plasma proteins (Fig. 5, Additional file 1: Figure S6). For Ga-FBP18, we observed high molecular weight species (70–100 kDa) in addition to low molecular weight metabolites, Fig. 5a. We identified Ga-transferrin as a metabolite by comparison with the retention time for a pure Ga-transferrin standard. Figure 5a also suggests that there may be other Ga-containing species with molecular weights less than transferrin, e.g. serum albumin. Interestingly we did not observe Ga-transferrin or other high molecular weight species when Ga-FBP17 was administered. The Ga-FBP17 result suggests that transchelation may be enhanced once the intact probe is degraded by proteases. For In-FBP19 injection (Fig. 5b), we did not identify In-transferrin in the plasma samples, but only observed the formation of low molecular weight degradation products.

## Discussion

The goal of this study was to assess the potential of HPLC-ICP-MS to characterize the metabolic behavior of imaging probes. A key question was whether we could detect probes at concentrations found in nuclear imaging studies. Previous nuclear imaging studies with fibrin binding peptides utilized a probe dose of about 1 nmol peptide. For fibrin binding peptides, 1–2% ID/g is typically observed in the blood a few minutes post injection, and with the sub-picomole limits of quantification determined here, HPLC-ICP-MS can quantify intact probe and metabolites at <0.1 %ID/g.

Each analysis run is preceded by a series of calibrant samples of varying concentration to generate a calibration curve. We used Ga(DOTA) and In(DOTA) complexes to avoid using large quantities of of peptide conjugate analyte for repeated calibration test runs and provide a generalizable protocol that allows for the quantification of any metabolite/ tracer molecule with the same calibrant. For ICP-MS analysis, (opposed to other MS methods), the metal ion is being quantified and not the intact molecule (or a fragment). Once the compound is nebulized onto the torch, the high temperature plasma combusts the sample to the atomic state. Thus although the DOTA complexes were used, the Ga/In ion rather than the complex is detected. Differences in ionization in the plasma can be further corrected by the use of an internal standard.

In order to look at metabolism in more detail, we employed a dose of ~100 nmol which allowed us to quantify metabolites down to 0.001 %ID/g. We selected two fibrin targeting peptide-chelator conjugates with known differential metabolic behavior and their corresponding gallium and indium complexes to test this methodology. Reverse phase and size exclusion chromatography revealed a wealth of metabolic information among the four probes tested. Moreover the metabolic profiles varied between the Ga- and In-labeled versions of the same peptide conjugate as has been seen in other systems (Blasi et al. 2014; Oliveira et al. 2015).

The ICP-MS detector offers many advantages over radiochemical detection. No radiation is required and the lack of radioactive decay means the samples can be frozen and analyzed at a later date if needed. Radiochemical detection requires relatively high amounts of activity for in-line detection, or samples can be collected and analyzed in a well counter, which decreases throughput and chromatographic resolution. Another attractive feature of ICP-MS is the ability to detect multiple mass labels nearly simultaneously. We showed here that Ga and In labeled probes could be co-injected and the plasma analyzed for metabolites. One could readily expand to using stable isotopes of the wide range of radionuclides used in nuclear medicine: Cu, Ga, In, I, Br, Al (for Al-F labeled probes), Zr, Lu, Y, Re, Bi, Sc, etc. In our example, we used peptides with the same chelator, but other combinations of peptide/chelator/label could be used.

There are some limitations to this work. We resolved some metabolites but no doubt more peaks could be resolved with further method development. However, the goal of the study was to assess the potential use of HPLC-ICP-MS to examine pharmacokinetics and metabolism of probes used in nuclear imaging, which we were able to do successfully. Similarly, identification of the individual metabolites was not attempted but this could be done with subsequent LC-MS-MS experiments. Additionally, incorporation of detection of radioactive species in tandem with the HPLC-ICP-MS system would provide an additional ideal validation step to compare retention times of radioactive and non-radioactive species. We used a standard HPLC, but a capillary HPLC would be expected to enable a much lower limit of quantification with very small sample volume required.

## Conclusion

We demonstrated that HPLC-ICP-MS is a useful method to screen imaging probes labeled with non-radioactive isotopes for pharmacokinetics and metabolism. Different columns (e.g. reverse phase and/or size exclusion) can provide a wealth of metabolic and transchelation information from small plasma sample volumes. The ability to detect multiple labels simultaneously allows the injection of multiple probes into the same animal thereby reducing animal numbers and rapidly identifying probes that may have suitable pharmacokinetic/metabolic behavior for further development.

## Additional file

Additional file 1: Figure S1. Supporting information is provided, including calibration curves for determination of LOD/LOQ, additional metabolite trace and quantification data using reverse phase and size exclusion. (DOCX 3329 kb)

## Abbreviations

DFO: Deferoxamine
DOTA: 1,4,7,10-Tetraazacyclododecane-1,4,7,10-tetraacetic acid
DOTP: 1,4,7,10-Tetraazacyclododecane-1,4,7,10-tetra(methylene phosphonic acid)
FBP: Fibrin binding peptide
HPLC-ICP-MS: High performance liquid chromatography inductively coupled plasma mass spectrometry
LC-MS: Liquid chromatography mass spectrometry
PET: Positron emission tomography
SEC: Size exclusion chromatography
SPECT: Single photon emission computed tomography
UV: Ultraviolet
UV/Vis: Ultraviolet/ Visible
