# Supplementary material for: Metabolite profiling with HPLC-ICP-MS as a tool for in vivo characterization of imaging probes
Source: EJNMMI Radiopharm Chem. 2018 Jan 22;3:2. doi: 10.1186/s41181-017-0037-5 (PMC5824709; doi:10.1186/s41181-017-0037-5)
Supplement: Supplementary file 1 — Supporting information is provided, including calibration curves for determination of LOD/LOQ, additional metabolite trace and quantification data using reverse phase and size exclusion. (DOCX 3329 kb) [file 41181_2017_37_MOESM1_ESM.docx]

Supporting Information for:

**Metabolite profiling with HPLC-ICP-MS as a tool for in vivo characterization of imaging probes.**

Eszter Boros,^1,3*^ Omar R. Pinkhasov,^1^ Peter Caravan^1, 2*^

^1^A. A. Martinos Center for Biomedical Imaging, Massachusetts General Hospital, Harvard Medical School, 149 13th Street, Suite 2301, Charlestown, Massachusetts 02129, United States

^2^ Institute for Innovation in Imaging, Department of Radiology, Massachusetts General Hospital, Boston, MA

^3^Current address: Department of Chemistry, Stony Brook University, 100 Nicolls road, Stony Brook, New York 11790, United States

**Correspondence:**

Peter Caravan, PhD, Building 149, Room 2301, 13th Street, Charlestown, MA 02129, USA. Phone: 617-643-0193, FAX: 617-726-7422, email: caravan@nmr.mgh.harvard.edu

Eszter Boros, PhD, Department of Chemistry, Stony Brook University, 100 Nicolls road, NY 11790, USA. Phone: 631-632 8572, email: eszter.boros@stonybrook.edu

**ORCID:** Eszter Boros: 0000-0002-4186-6586

Peter Caravan: 0000-0002-3179-6537

Reverse phase calibration curves:

Figure S1. Probe calibration curves generated for reverse phase method using Ga(DOTA) and In(DOTA) complexes (top panel). Examples of HPLC traces for Ga(DOTA) and In(DOTA) used for this calibration are provided within the bottom panel.

Assessment of detection limits:

**Table S1.** Limit of blank (LOB), limit of detection (LOD) and limit of quantification (LOQ) for Ga and In were determined using the standard deviation of five blank (no Ga or In content) and five low concentration samples (1.056 pmol for Ga(DOTA), 0.85 pmol for In(DOTA)). LOD and LOQ were determined using established methods(*1*): LOD = S_b_ +3* SD_Sb_ (blank), LOQ = S_b_+10*SD_Sb_. (S_b_ = average signal of blank; SD_Sb =_ standard deviation of blank).

|  | MacDougall et al.(*1*) |
| --- | --- |
| LOD (Ga) | 0.533 pmol |
| LOD (In) | 0.165 pmol |
| LOQ (Ga) | 0.885 pmol |
| LOQ (In) | 0.459 pmol |

Synthesis and purification of probes:

**Table S2.** Mass spectrometric characterization summary of complexes utilized for this work.

| Compound | Formula | Theoretical M_W_ | Observed M_W_ | Retention time |
| --- | --- | --- | --- | --- |
| ^nat^Ga_2_FBP17 | C_103_H_142_ClGa_2_N_25_O_29_S_2_ | 2432.81 Da | 1217.9 [M + 2H]^2+^ | 15.3 min |
| ^nat^In_2_FBP15 | C_103_H_142_ClIn_2_N_25_O_29_S_2_ | 2522.76 Da | 1262.9 [M + 2H]^2+^ | 16.1 min |
| ^nat^Ga_2_FBP18 | C_106_H_141_ClGa_2_N_24_O_30_S_2_ | 2513.77 Da | 1257.3 [M + 2H] ^2+^ | 15.7 min |
| ^nat^In_2_FBP19 | C_106_H_141_ClIn_2_N_24_O_30_S_2_ | 2559.75 Da | 1280.8 [M + 2H]^2+^ | 15.2 min |


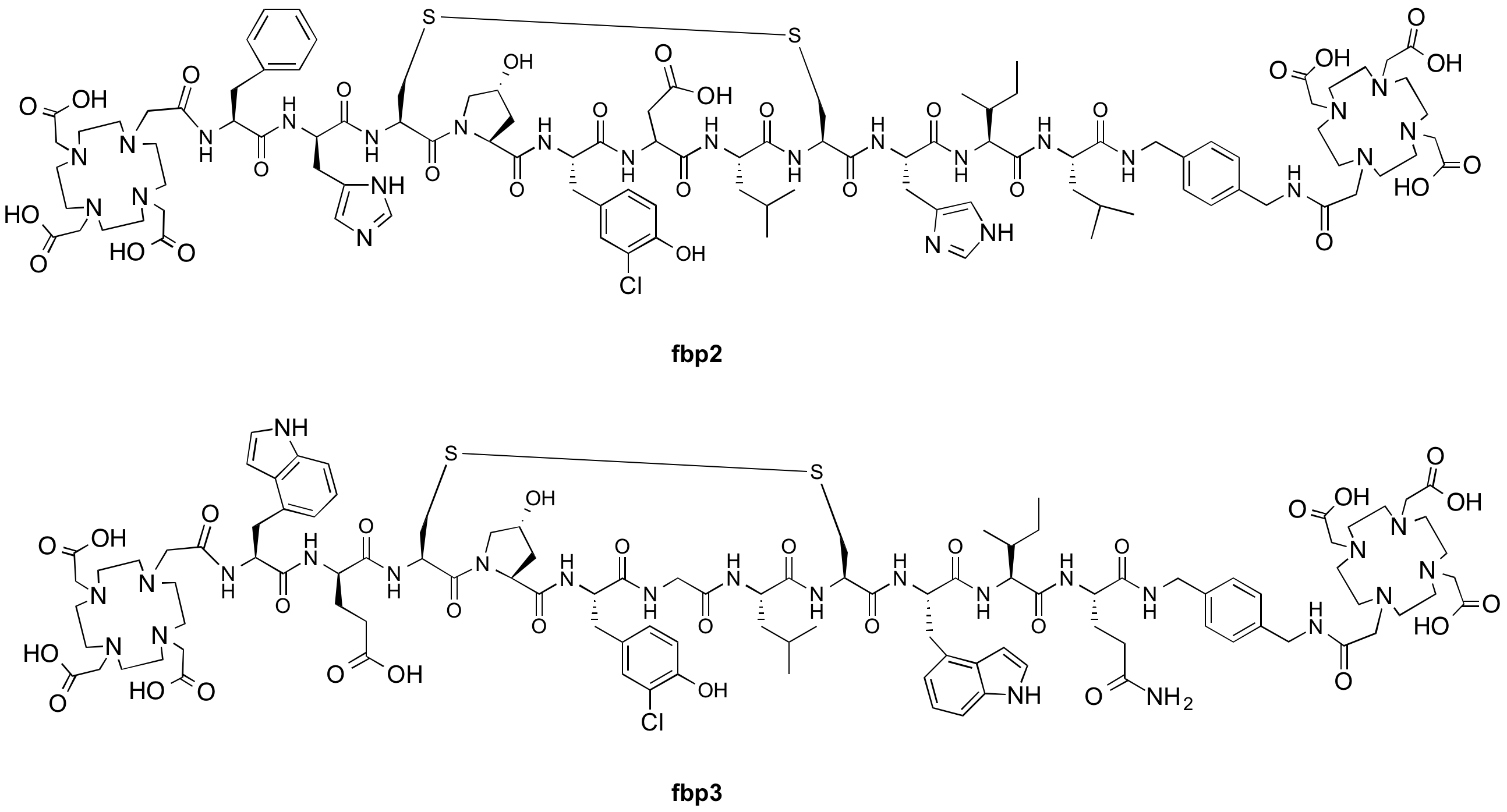


Figure S2. Chemical structures of uncomplexed DOTA-peptide conjugates.

RP and SEC assessment of blood samples:

Sampling and detection was carried out as described in the main portion of the manuscript.

Figure S3. Overlays of plasma sample analysis using reverse phase chromatography from blood draws at 0, 5, 10, 15, 30 and 60 minutes post injection. Grey bar denotes peak of intact probe.

Figure S4. Quantification of total probe, as well as individual metabolite groups using blood plasma samples obtained at each time point and reverse phase chromatography for compounds GA-FBP17 and In-FBP15.

Figure S5. LC-ICP SEC analysis of Ga-transferrin and In-transferrin samples. Both sulfur and gallium/indium detection are shown to confirm formation of multiple transferrin species.

Figure S6. Overlays of plasma sample analysis using size exclusion chromatography from blood draws at 0, 5, 10, 15, 30 and 60 minutes post injection. Orange bar denotes Ga-transferrin adduct, grey bar denotes peak of intact probe.


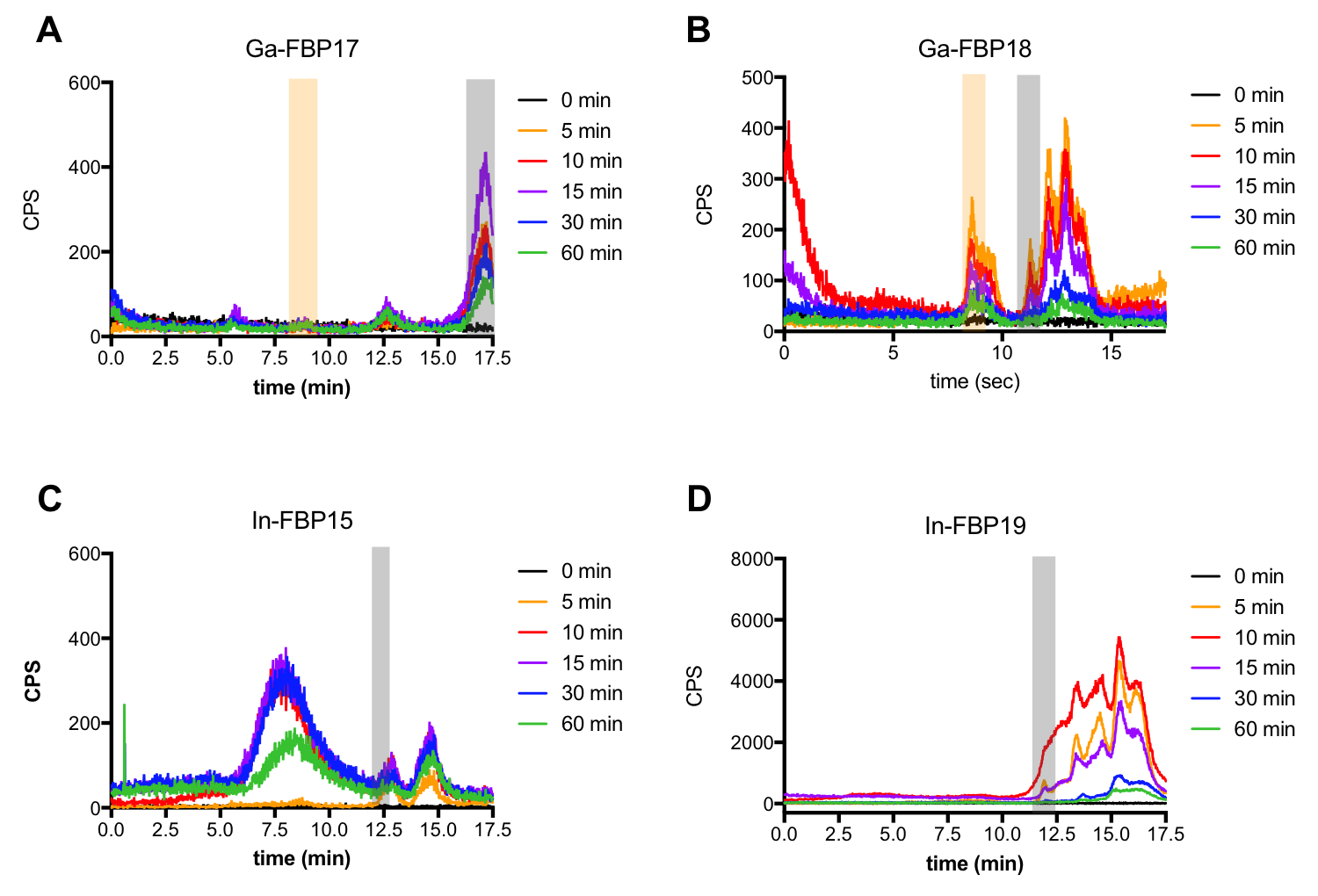


Figure S7. Quantification of intact probe at 5, 10, 15, 30 and 60 minutes post injection. Filled symbols represent results obtained from single probe injection; open symbols show results from co-injection experiments (Ga-FBP17/In-FBP15).


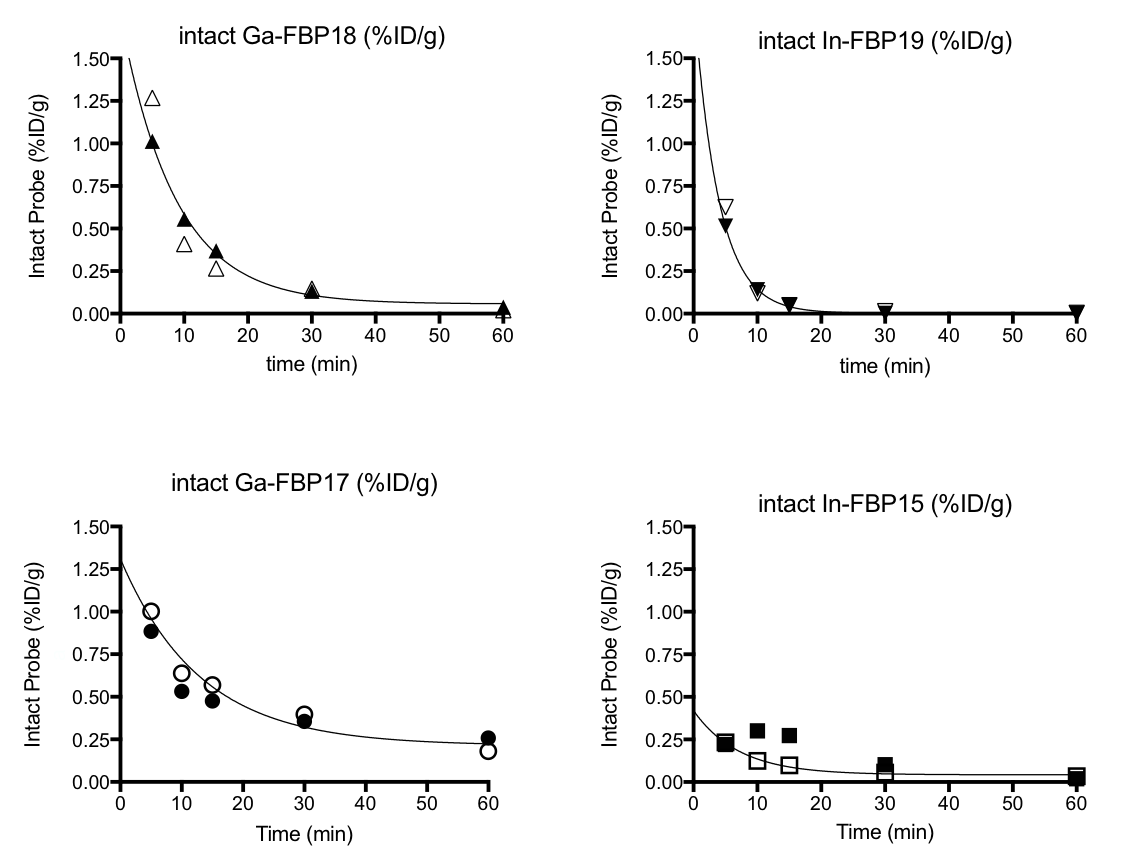


References:

**1.** Armbruster DA, Pry T. Limit of Blank, Limit of Detection and Limit of Quantitation. *Clin Biochem Rev.* 2008;29:S49-S52.

**2.** MacDougall D, Crummett WB. Guidelines for data acquisition and data quality evaluation in environmental chemistry. *Anal Chem.* 1980;52:2242-2249.

**3.** Oliveira BL, Blasi F, Rietz TA, Rotile NJ, Day H, Caravan P. Multimodal molecular imaging reveals high target uptake and specificity of 111In-and 68Ga-Labeled fibrin-binding probes for thrombus detection in rats. *J Nucl Med.* 2015;56:1587-1592.

**4.** Ciesienski KL, Yang Y, Ay I, et al. Fibrin-Targeted PET Probes for the Detection of Thrombi. *Mol Pharm.* 2013;10:1100-1110.
